# Iranian nurses’ perspective of barriers to sexual counseling for patients with myocardial infarction

**DOI:** 10.1186/s12912-021-00697-x

**Published:** 2021-10-13

**Authors:** Parvin Mangolian Shahrbabaki, Roghayeh Mehdipour-Rabori, Tayyebeh Gazestani, Mansooreh Azzizadeh Forouzi

**Affiliations:** 1 Nursing Research Center, Kerman University of Medical Sciences, Kerman, Iran; 2 Razi Faculty of Nursing and Midwifery, Department of Critical Care Nursing, Kerman University of Medical Sciences, Kerman, Iran; 3 Razi Faculty of Nursing and Midwifery, Department of Medical- Surgical Nursing, Kerman University of Medical Sciences, Kerman, Iran; 4 RN and MS of Medical-Surgical Nursing, Seyed Shohada Hospital, Kerman, Iran; 5 Neuroscience Research Center, Institute of Neuropharmacology, Kerman University of Medical Sciences, Kerman, Iran

**Keywords:** Sexual counseling, Myocardial infarction, Barriers, Nurse

## Abstract

**Background:**

Sexual counseling is an essential part of cardiac rehabilitation. This study aimed to investigate Iranian nurses’ perceptions of barriers to sexual counseling for patients with myocardial infarction.

**Methods:**

This cross-sectional study included 169 nurses who worked in CCUs, Post CCUs, and cardiac surgery wards of teaching hospitals in southeastern Iran. The barriers to providing sexual counseling inventory was used to assess sexual counseling barriers for patients with myocardial infarction. SPSS 19 was used to analyze the data. The significance level was 0.05. This study lasted from November 2019 to March 2020.

**Results:**

The results showed that the highest mean scores for patient-related barriers were related to the patient’s religion and belief (2.83 ± 0.52) and embarrassment (2.82 ± 0.52 %). The highest scores for nurse-related barriers were related to nurse’s discomfort in discussing sexual issues (2.67 ± 0.62), a lack of experience in sexual counseling (2.62 ± 0.65), and sexual hesitation in advising patients (2.57 ± 0.7). The highest mean scores for organizational barriers were a lack of managerial attention and support for counseling (2.67 ± 0.66) and a lack of proper supervision system (2.62 ± 0.72).

**Conclusions:**

Religion/beliefs, embarrassment and a lack of managerial attention and support for counseling were the most important barriers in the organizational, nursing and patient domains. Since sexuality is a significant issue in most cultures and religions, particularly in Islamic countries, health care professionals should work to change the attitudes of patients towards sexuality through sexual health education and counseling to meet patients’ needs and improve their sexual health.

## Introduction

Sexuality is a health determinant that has a positive effect on both physical and mental health [1]. Sexual problems have a widespread impact on quality of life and can have negative consequences for emotional health and interpersonal relationships [2]. Sexual health is defined as a state of physical, mental, and social well-being in relation to sexuality [3]. Many diseases, including heart disease, have many negative effects on different aspects of patients’ lives, including sexual activity [4, 5].

Patients with myocardial infarction experience many sexual concerns [6] and a large number of them limit or stop their sexual activity for fear of re-myocardial infarction or sudden death [7, 8]. Returning to sexual activity after myocardial infarction and achieving sexual satisfaction are difficult for patients and their spouses [9]. Given the positive effects of treatment for sexual problems, the healthcare team should consider early detection and treatment of sexual issues [10]. A counseling program should be considered as a part of routine care for cardiovascular patients [9, 11].

Sexual counseling is interaction with the patient to obtain information about sexual concerns and a safe return to normal sexual activity. Assessing, supporting, and providing specific information on psychosexual problems is also a part of sexual counseling [12, 13]. As spouses play an important role in cardiac rehabilitation [14], nurses have the opportunity to interact with patients and their spouses and identify their sexual needs in order to achieve optimal well-being [1]. Nurses, on the other hand, play an important role in providing lifestyle education for cardiac patients [15]. However, some of them do not consider the exchange of information about sexual concerns of the patients with myocardial infarction [16]. According to reports, most nurses do not consider cardiac patients’ sexual needs as an essential part of their assessment, do not routinely assess their sexual problems, and are reluctant to do so [9, 17].

According to the literature review, there are numerous barriers to providing sexual counseling to patients. According to a Turkish study, almost all nurses (99.4 %) did not provide sexual education to patients with myocardial infarction due to a lack of knowledge and skill, as well as their belief that sexual issues were a patient’s private domain [18]. A qualitative systematic review in Pakistan showed that institutional barriers such as lack of education, guidelines, and material resources, personal barriers such as giving least priority to sexual counseling and lack of comfort with discussing sexuality, and socio-cultural and religious barriers such as contradictory beliefs and gender differences canchallenges nurses when providing sexual education to patients with myocardial infarction [19]. A literature review in Jordan on barriers to sexual health assessment for patients with coronary artery diseases in nursing practice showed that barriers to addressing sexual health are multifactorial and were related to patients, nurses, organization, and value [20]. It seems that people of different racial groups demonstrate different patterns of sexual dysfunction which are attributed to the different racial, ethical, cultural and customary factors in different communities [21].

Sexual activity outside of marriage is illegal in Iran, and the only socially acceptable type of sexual relationship is a monogamous relationship. There is little information available in Iran on the prevalence of sexual difficulties. Estimating the prevalence is difficult due to socio-cultural barriers, taboos, and misconceptions. According to Sayyadi et al. (2019), Iranian people avoid discussing issues related to marital relations due to shame and modesty [22]. According to one study (n = 350), 93.1 % of women and 80.6 % of men reported some form of sexual dysfunction [23]. Another study in sexual script section demonstrated that Iranian women’s sexual behaviours were affected by a sexual script that was affected by religious instructions and traditional attitudes that are partially wrong [24]. Other researchers in Iran concluded that religious teachings have a basic role in sexual health of women. However, occasional inconsistency between beliefs, learning and sexual expectations, practices, and situations would lead to jeopardize the psychological and somatic health of women [25]. A qualitative study revealed that women’s sexual self-understandings and their sexual behaviors are strongly determined by “androcentricity”, this being relevant both to sexuality education and reproductive health [26]. Mohammadian and Dolatshahi showed that 77.6 % of women and 35.6 % of men reported sexual problems (n = 1129) [27]. It has been shown that marital satisfaction rates among Iranian coronary artery disease patients are lower than in the general population with 62.22 % of these patients having significant sexual problems [28]. Another study found that most Iranian patients with myocardial infarction had at least one problem with their sexual function [28]. It seems that cultural contexts, lack of education and sexual counseling, poor partner techniques are the most important causes of sexual dysfunction in Iranian literature [11, 22, 27–30]. Using a qualitative approach, Farahani et al. (2008) found a variety of cultural factors influencing patient education, including patients’ lifestyle, beliefs in disease and treatment, concealment of true diagnosis, different opinions regarding the preferred instructor, and ineffective communication [31]. From the perspective of Iranian cardiologists, the most common barriers to discussing sexual issues include the patient’s discomfort in discussing sexual issues, religious and cultural reasons, lack of time, the presence of another person, and lack of knowledge and skills in dealing with sexual issues [11]. Some nurses believe that assessing patients’ sexual needs is taboo [32]. Sex therapists in Iran provide sexual counseling services in sexual health clinics, family health clinics, and psychological clinics. A sex therapist is a person who specializes in treating sexual dysfunction. To address the sexual health needs of patients, Iranian nurses must be aware of the sexual needs of patients with myocardial infarction and refer them to sex therapists. To meet the needs of patients, awareness and identification of the sexual needs and when to refer to a specialist will help to ensure this area of practice alongside physical and mental health [1].

Given the above, it appears that sexuality has particular importance because these issues are profoundly affected by culture, beliefs, social background, and also family beliefs [21]. This research was conducted in response to the fact that most sexual health studies in Iran have been conducted on the general population or patients with chronic diseases whereas studies on acute diseases such as myocardial infarction are limited. On the other hand, the existing barriers have not been classified as being related to the patient, the nurse, and the organization. In addition, sexual behavior and performance have a significant effect on the quality of personal and marital life [11]. The recognition of these barriers will provide new information that will be beneficial in future planning and intervention for improving the sexual health care of cardiovascular patients. Therefore, the current study aimed to investigate the barriers to sexual counseling for patients with myocardial infarction from the perspectives of nurses working in cardiac care units in hospitals located in southeastern Iran.

## Methods

### Study design and setting

This study had a cross-sectional descriptive-analytical design. The study settings included CCUs, Post CCUs, and cardiac surgery units of three teaching hospitals affiliated to Kerman University of Medical Sciences, which are the largest centers in southeastern Iran. This study lasted from February to May 2020.

### Sample size and sampling

The study population consisted of 180 nurses working in CCUs, Post CCUs, and cardiac surgery wards in 2019. Inclusion criteria were having a diploma or above degrees in nursing and a history of caring for patients with heart diseases. One hundred and sixty-nine nurses participated in this study, so the response rate was 93.88 %.

### Instruments

Two questionnaires were used to collect data on demographic characteristics and barriers to providing sexual counseling for patients with myocardial infarction. Demographic and background information included age, sex, marital status, education, ward, work experience, and length of time working with cardiac patients, and the level of information about sexuality.

The questionnaire on barriers to sexual counseling for patients with myocardial infarction was created by a researcher and based on the literature review [19, 20, 33]. It consisted of 30 items divided into three domains of patient-related barriers (13 items), nurse-related barriers (12 items), and organizational barriers (5 items). A three-point Likert scale was used (agree = 3, no idea = 2, disagree = 1). We used the mean score, with one being the lowest possible score and three being the highest possible score, and high scores indicating a better perception of barriers. To determine the content validity of the questionnaire, it was distributed to expert faculty members from the school of nursing (n = 10). After gathering the opinions of professors, the content validity index (CVI) was determined to be 0.80 and the content validity ratio (CVR) was 0.79. The Cronbach’s alpha coefficient in the current study was 0.78 for the total scale, 0.79 for the patient-related barriers, 0.83 for nurse-related barriers, and 0.79 for the organizational barriers.

### Data collection

Data collection began after obtaining a code of ethics from the Ethics Committee of Kerman University of Medical Sciences. The first researcher visited different wards of each hospital in different shifts. After coordinating with managers and explaining the aims of the study to the nurses, she provided the questionnaire to the participants when they were physically, mentally ready to participate in the study. She delivered the questionnaire from the staff at the same or subsequent shifts. The participants were explained about information confidentiality. Instead of names, participant codes were used to label data and a separate list of code-to-name match-ups was kept. Only the participant code was used when recording or publishing the data. We were also careful not to publish enough information so that the participants could be identified.

### Data analysis

Data were analyzed using SPSS 18. Descriptive statistics (frequency, percentage, mean, and standard deviation) were used to describe data. Independent t-test and ANOVA were used to evaluate the mean scores of barriers to sexual counseling in terms of background characteristics. The significance level was 0.05.

## Results

The mean age of nurses was 36.12 ± 8.06 years. Most of the nurses were female (91.1 %), married (84.9 %), and had a bachelor’s degree (91.7 %). The mean work experience of nurses in cardiac care units was 8 years. The study included 74 % of the nurses in CCUs, 13 % in post-CCUs, and 10.7 % in cardiac surgery units. The majority of nurses (99.4 %) had not completed any specialized or retraining courses on sexual counseling for cardiac patients and believed in the low number of courses as well as limited training about sexual counseling (Table 1).

**Table 1:** Demographic and background characteristics of nurses in cardiac care units

Variable		M	SD
Gender	Female	154	91.1
	Male	15	8.9
Marital status	Single	25	15.1
	Married	143	84.9
Level of education	BSc	155	91.7
	MSc	14	8.3
Position	Nurse	154	91.1
	Head nurse	15	8.9
Ward	CCUs	125	74
	Cardiac ward	22	13
	Cardiac surgery	18	10.7
Pass specialized training and retraining	Yes	1	0.6
	No	168	99.4
The courses spent about sexual counseling patients	High	1	0.6
	Average	10	5.9
	Low	51	30.2
	Very low	107	63.3
The internship spent about sex advice	High	1	0.6
	Medium	5	3
	Low	40	23.7
	Very low	123	72.7
**Variable**	**M**	**SD**	
Age	36.12	8.06	
Work experience	12.12	7.57	
Experience in cardiac surgery	8.36	7.18	

The highest mean scores related to the barriers to sexual counseling for patients with myocardial infarction were related to patients (33.96 ± 57.5), nurse (29.47 ± 4.95), and organization (12.86 ± 2.65), respectively (Table 2).

**Table 2:** Barriers to sexual counseling in patients with myocardial infarction (related to Patient)

Statements related to barriers	agree		Disagree		No idea		score	
	N	%	N	%	N	%	M	SD
Short hospital stay	89	52.7	44	26	36	21.3	2.31	0.80
Lack of time for training	91	53.8	60	35.5	18	10.7	2.43	0.68
Cultural differences between patient and nurse	139	82.2	17	10.1	13	7.7	2.75	0.59
Patient’s unwillingness to discuss sexual issues	129	76.3	18	10.7	22	13	2.63	0.70
Having or not having a companion	82	48.5	47	27.8	40	23.7	2.25	0.82
Social status of the patient	132	78.1	21	12.4	16	9.5	2.69	0.64
Patient age	127	75.1	17	10.1	25	14.8	2.60	0.73
Patient distrust of nurse to discuss sexual concerns	117	69.2	24	14.2	28	16.6	2.53	0.76
Fear of confidentiality	131	77.5	21	12.4	17	10.1	2.67	0.65
The veil of patients	148	87.6	12	7.1	9	5.3	2.82	0.50
Religious and religious reasons for the patient	151	89.3	7	4.2	11	6,5	2.83	0.52
Not asking the patient about sexual issues.	143	84.6	12	7.1	14	8,3	2.76	0.59
Patient’s negative attitude about discussing sex	138	81.7	8	4.7	23	13.6	2.68	0.70
**Total score (Barriers related to the patient)**							**33.96**	**5.57**

The highest mean scores for patient-related barriers were related to patients’ religion and belief (2.83 ± 0.52) and embarrassment (2.82 ± 0.52). The highest mean scores for nurse-related barriers were related to the nurses being uncomfortable discussing sexual issues (2.67 ± 0.62), a lack of experience in sexual counseling (2.62 ± 0.65), and sexual hesitation in advising patients (2.57 ± 0.7) (Table 3). The highest mean scores for organizational barriers were related to a lack of managerial attention and support for counseling (2.67 ± 0.66) and a lack of proper supervision system in the field of sex counseling (2.62 ± 0.72) (Table 4).

**Table 3:** Barriers to sexual counseling in patients with myocardial infarction (related to nurses)

Statements related to barriers	agree		Disagree		No idea		score	
	N	%	N	%	N	%	M	SD
High density of nursing duties	108	63.9	40	23.7	21	12.4	2.51	0.71
Lack of time for patient education	106	62.7	44	26.1	19	11.2	2.51	0.69
Lack of proper communication between the nurse and the patient	100	59.2	51	30.1	18	10.7	2.49	0.68
Lack of nurse motivation for counseling	86	50.9	53	31.3	30	17.8	2.33	0.76
Nurses’ disbelief on the impact of counseling on treatment	59	34.9	79	46.8	31	18.3	2.17	0.71
Additional processes such as administrative hierarchy	84	49.7	49	29	36	21.3	2.28	0.80
Nurses are not comfortable talking about sex	126	74.6	30	17.7	13	7.7	2.67	0.62
Lack of nurse knowledge on sex issues	100	59.5	47	28	21	12.5	2.47	0.71
Doubt about providing sexual counseling to the patient	117	69.3	32	18.9	20	11.8	2.57	0.70
Lack of experience in sex counseling	121	71.6	32	18.9	16	9.5	2.62	0.65
Uncertainty about the ability to have sex counseling	89	53.3	50	29.9	28	16.8	2.37	0.76
Nurses’ Cultural Beliefs	110	65.5	32	19	26	15.5	2.50	0.75
**Total score (Barriers related to the nurses)**							**29.47**	**4.95**

**Table 4:** Barriers to sexual counseling in patients with myocardial infarction (related to organization)

Statements related to barriers	agree		Disagree		No idea		score	
	N	%	N	%	N	%	M	SD
Lack of facilities for patient privacy	104	61.5	47	27.8	18	10.7	2.51	0.68
Section restrictions on discussing sexuality	106	62,7	45	26.6	18	10.7	2.51	0.68
Lack of proper environment for consultation	114	67.5	33	19.5	22	13	2.54	0.72
Lack of attention and support from managers for counseling	132	78.1	19	11.2	18	10.7	2.67	0.66
Lack of proper monitoring and feedback system on sexual counseling	128	75.7	17	10.1	24	14.2	2.65	0.72
**Total score (Barriers to organization)**							**29.47**	**4.95**

Based on the Independent t-test and ANOVA, results related to the relationship between demographic variables and scores of barriers to sexual counseling (barriers related to patient, nurse, and organization) showed only a significant correlation between nursing education level and organizational barriers (*P* = 0.015). Therefore, postgraduate nurses got higher mean scores in organizational barriers. When it came to discussing sexuality, there was not a difference between males and females and between the three groups (CCU, post CCU and cardiac surgery departments).

## Discussion

The results showed that the patient’s religion \ belief and embarrassment had the highest mean scores in patient-related barriers. The highest mean scores in nurse-related barriers were related to nurse’s discomfort in discussing sexual issues, lack of experience, and sexual hesitation in counseling patients. The highest mean scores in organizational barriers were a lack of managerial attention and support for counseling and a lack of proper supervision system about sex counseling.

The results showed most barriers were related to the patient domain; most patients do not want to receive sexual counseling. Therefore, nurses were unable to begin sexual discussions. Based on studies, discussing sexuality is still subject to uncertainty and sometimes fear of being judged. Patients will be the main barrier to counseling if they do not want to talk about it[11, 20]. Consistent with these results, studies found that factors influencing patient education were related to patients’ lifestyle and beliefs in disease and treatment [31, 34, 35]. Culture, according to researchers, influences beliefs and attitudes toward sexual well-being [1]. Recognition of culture will facilitate understanding of the cultural foundations of sexuality help health providers in suggesting culturally appropriate and compatible forms of health care [26]. However, another study in the Netherlands showed that most barriers were related to the organizational dimension, with the absence of organizational policies being the most significant obstacle to sexual counseling [33]. These differences can be attributed to differences in culture, beliefs, social background, and organizational facilities [31]. Cardiologists, nurses, and other medical staff can use the PLISSIT model in clinical centers to assess the sexual needs, weaknesses, and strengths of the affected patients because the PLISSIT model can help patients improve their quality of sexual life and sexual functioning [1].

Religion and beliefs, according to nurses, were the most significant barriers to providing sexual counseling for patients with myocardial infarction. There are some misconceptions about religious beliefs. Sexual issues are taboo for some Iranians and they believe that these issues in religion are bad and polluted. Families with a strong religious upbringing who constantly intimidate the opposite sex have difficulty discussing sexual issues [1]. Some studies found that culture had an impact on sexual counseling [14, 33]. According to studies, 94 % of nurses prohibited from discussing sex [32]. Two studies in Turkey have emphasized that religion and culture were identified as barriers to patients’ sexual assessment [36, 37]. The researchers argue that traditional religious values lead to shame about sexual issues. They emphasize that religious beliefs vary across cultures because sex is a personal matter that is difficult to discuss [1, 38]. A study demonstrated that religion was a key factor in women’s sexual self-understanding; in addition they noted sexual obedience has a great impact on women’s chastity and self- steam [24]. Other study revealed that religious-related misconceptions have essential role in creating sexual problems [25].

Embarrassment was another patient-related barrier. Several studies have found that shame and embarrassment are the barriers to sexual counseling in different countries and patients are unwilling to discuss it [39, 40]. Another study reported that sexual counseling for patients with heart failure was a silent phenomenon. They also found that German nurses rarely provided sexual counseling for patients [41]. Patients, particularly Iranian ones, may feel embarrassed about sexuality, especially sexual intercourse, and may be reluctant to discuss or even respond to such questions [1, 31]. Based on a study, patients were not interested in discussing sex because of their embarrassment, so they should be prepared in this regard [33]. These findings highlight the importance of professional intervention. We believe it should focus on minimizing the individual’s feelings of shame. Nurses can prepare the patients by providing guidance on sexual activity after MI at the time of admission and asking patients to read the guidelines carefully throughout their hospitalization. Providing a safe environment in which patients can consensually discuss issues related to sex and sexuality after MI also prepares patients to counsel with the nurses. The PLISSIT model can be used in conjunction with medical treatment to help improve the quality of sexual life and sexual functioning in patients with myocardial infarction by teaching coping and problem-solving skills and encouraging participation in group programs for expressing feelings and attitudes about one’s current sex life.

The results showed that nurses who were skeptical about the impact of sexual counseling on the treatment process received the lowest mean score. No similar study was found, but nurses are aware of the variety of their roles, including assisting patients with sexual concerns [30, 34]. Ozdemir and Akdemir (2008) indicated that the nurses did not provide sexual education due to a lack of knowledge, confidence, and skill. Another study showed the barriers such as putting sexual counseling last on the priority list and being uncomfortable discussing sexuality can make it difficult for nurses to provide sexual education to patients [18]. A nurse acting as a counselor can help a patient to deal with changes and problems, as well as cope with new conditions. The researchers believe that healthcare personnel should be more involved in the sexual health of patients [42].

Regarding the relationship between nurses’ demographic variables and barriers related to sexual counseling, there was a significant correlation only between the nursing education level and organizational barriers. Therefore, postgraduate nurses assigned higher scores to organizational barriers, and they could provide counseling and better deal with patient and nurse barriers while dealing with organizational barriers is probably more complicated. A study showed that paying attention to sexual health problems during undergraduate education could increase their knowledge [20].

There were some limitations to this study. First, there was only one measurement method used in this study, which was a self-report questionnaire. Second, because this is a cross-sectional design, and the researcher cannot conclude cause and effect; therefore, the interventional study would be helpful to remove barriers of sexual counseling. Third, a researcher- made questionnaire that its psychometric properties have not be studied was used in this study. Fourth, because the number of participants was small, generalizing across populations would be inappropriate.

## Conclusions

To the best of our knowledge, this is the first study in Iran to examine cardiac nurses’ perception of barriers to sexual counseling for patients with myocardial infarction. This information is essential for the development of sexual health in cardiac patients. Nurses who participated in this research identified several barriers to providing sexual counseling, which must be reported for the improvement of sexual counseling interventions.

Religion/beliefs and embarrassment were more important barriers in Iran than in other countries. Since sexuality is a sensitive issue in most cultures and religions, particularly in Islamic countries, health policymakers should intervene to raise awareness and change the attitude of people to reduce the sensitivity of the community and gradually remove this taboo. It is also recommended that depending on the educational needs of patients, nurses provide primary sexual health guidance and trained counselors provide specialized counseling. Referring patients to a specialized sexual health clinic in a hospital or trained counselors also can help to remove barriers. Providing sexual counseling programs for nurses working in the cardiac department may also be beneficial in decreasing barriers to sexual counseling. The final solution and recommendation to remove the barriers identified in this study is to investigate these barriers and develop appropriate interventions, as it is evident, nurses encounter many barriers when providing sexual counseling to patients. In addition, Cardiologists, nurses, and other medical staff can use coping and problem-solving skills and encourage participation in group programs for expressing feelings and attitudes towards one’s current sexual life.

## Data Availability

Data are available by contacting the corresponding author.
